# Pluripotent Stem Cell Studies Elucidate the Underlying Mechanisms of Early Embryonic Development

**DOI:** 10.3390/genes2020298

**Published:** 2011-03-24

**Authors:** Lingyu Li, Naihe Jing

**Affiliations:** Laboratory of Molecular Cell Biology, Institute of Biochemistry and Cell Biology, Shanghai Institutes for Biological Sciences, Chinese Academy of Sciences, 320 Yue Yang Road, Shanghai 200031, China; E-Mail: lyli@sibs.ac.cn

**Keywords:** early embryonic development, BMP, FGF, Nodal, Wnt, ESCs, EpiSCs

## Abstract

Early embryonic development is a multi-step process that is intensively regulated by various signaling pathways. Because of the complexity of the embryo and the interactions between the germ layers, it is very difficult to fully understand how these signals regulate embryo patterning. Recently, pluripotent stem cell lines derived from different developmental stages have provided an *in vitro* system for investigating molecular mechanisms regulating cell fate decisions. In this review, we summarize the major functions of the BMP, FGF, Nodal and Wnt signaling pathways, which have well-established roles in vertebrate embryogenesis. Then, we highlight recent studies in pluripotent stem cells that have revealed the stage-specific roles of BMP,FGF and Nodal pathways during neural differentiation. These findings enhance our understanding of the stepwise regulation of embryo patterning by particular signaling pathways and provide new insight into the mechanisms underlying early embryonic development.

## 1. Introduction

Embryogenesis is a process by which the zygote develops into a complex and organized embryo. During early development in the mouse, the zygote first develops into a blastocyst containing the inner cell mass (ICM) inside the trophectoderm. At embryonic day (E) 4.5, the blastocyst implants in the uterus and the ICM differentiates to form the primitive endoderm and the early epiblast [1]. At E5.5, the early epiblast develops into a columnar epithelial monolayer of pluripotent cells called the late epiblast [2]. At approximately E6.5, gastrulation commences with the formation of the primitive streak in the posterior of the epiblast. Epiblast cells that ingress through the primitive streak form the mesoderm and endoderm. The cells remaining in the anterior of the epiblast form the ectoderm [3]. As development proceeds, the ectoderm, mesoderm and endoderm generate most of the cell types and organs of the adult.

For studies of early development, *Xenopus*, zebrafish and chick embryos are widely used, as they are easily raised and can be manipulated with various assays. Compared with these species, mice as mammals more closely resemble humans in developmental processes, but mouse embryos are difficult to manipulate, especially after implantation in the uterus. In past years, genetic, molecular and cell transplantation experiments in these animal models have established that the BMP, FGF, Nodal and Wnt signaling pathways play crucial roles in early embryonic patterning. However, there have been inconsistent results obtained from *Xenopus*, zebrafish and chick, probably caused by species-specific differences or by interference from complex germ-layer interactions. In the mouse embryo, knowledge of the molecular mechanisms governing early embryogenesis is limited due to the small size, complexity and inaccessibility of the early post-implantation embryo. Thus, mechanistic studies require an amenable system with embryonic properties but with the absence of the complexity that exists *in vivo*. Under these circumstances, pluripotent stem cells derived from different developmental stages have become an *in vitro* system for investigating the detailed molecular mechanisms through which signaling pathways regulate cell fate decisions. Both human and mouse embryonic stem cells (ESCs) are pluripotent cell lines derived from blastocyst-stage embryos [4–7]. Under appropriate culture conditions, ESCs differentiate into derivatives of all three germ layers, and the differentiation of specific cell types from ESCs is directed by a set of signals similar to that which regulates embryonic development *in vivo* [8–11]. Recently, another type of pluripotent stem cell, referred to as epiblast stem cells (EpiSCs), was derived from the late epiblast tissue of E5.5 mouse embryos. EpiSCs are molecularly and epigenetically distinct from mouse ESCs, but they share characteristics with human ESCs [12–14]. ESCs, corresponding to the ICM or the early epiblast state, combined with EpiSCs, which represent an *in vitro* equivalent of the late epiblast, form a novel system for studying the mechanisms of early embryonic development, especially for mechanistic studies at different developmental stages. In this review, we briefly summarize the key functions of the BMP, FGF, Nodal and Wnt signaling pathways in early embryogenesis, and then we discuss recent findings obtained from studies in ESCs and EpiSCs that reveal the stage-specific functions of BMP, FGF and Nodal signals. These findings begin to elucidate the mechanisms underlying different stages of early embryonic development.

## 2. BMP Signaling

The bone morphogenetic proteins (BMPs) are members of the transforming growth factor β (TGF-β) cytokine superfamily. BMP signaling has been shown to play a central role in ectodermal cell fate decisions. Using ectodermal explants (also called animal caps) from *Xenopus* blastula-stage embryos, researchers have shown that activation of the BMP pathway in ectoderm leads to the acquisition of an epidermal fate, whereas inhibition of BMP signaling by antagonists that are secreted by the Spemann organizer leads to a neural fate [15,16]. These results suggested that the ectoderm has a natural “default” tendency to differentiate into neural tissues unless it is instructed by BMP to become epidermis [17]. Since the “default model” was proposed, there has been debate concerning whether BMP inhibition is adequate for neural induction, because opposing results have been obtained with different assays in the chick. Initially, it was shown that grafts of cells expressing BMP4 or BMP7 failed to inhibit neural plate formation [18]. However, in epiblast explants from chick embryos, BMP4 showed a capacity to inhibit neural fate and promote an epidermal fate [19]. Furthermore, electroporation of BMP4 into the prospective neural plate inhibits the expression of the definitive neural markers (*Sox2* and late *Sox3*), but it does not affect the pre-neural marker (early expression of *Sox3*). Therefore, BMP inhibition is probably required only as a late step during neural induction [20].

Other data relevant to the default model come from experiments showing that BMP antagonists are unable to induce neural character in the epidermal or extra-embryonic ectoderm of chick embryos [20–22]. Moreover, single or double BMP antagonist (*chordin* and *noggin*) mouse mutants show relatively little change in the initial size of the neural plate [23–25]. These results suggest that BMP inhibition is not sufficient to induce neural cells. The default model was not confirmed in mice until the generation of BMP receptor (*Bmpr1a*) knockout mice. Bmpr1a is the only type I BMP receptor expressed in the epiblast of implanted mouse embryos [26]. Knockout of *Bmpr1a* in mouse embryos, which completely inhibits BMP activity, was found to lead to premature neural differentiation of the epiblast accompanied by suppression of mesodermal fate [27]. Therefore, BMP inhibition is essential for neural differentiation in mice. However, the possibility that some other signals participate in neural induction cannot be excluded. That BMP inhibits neural differentiation continually in the epiblast rather than at a specific time point has been proposed [27].

The discrepancies of the data obtained from chick and mouse raise important questions, including whether there is a time point during which the BMP signal inhibits neural induction and what mechanisms are involved in this process. These questions are difficult to answer using only *in vivo* studies. Recently, some findings in pluripotent stem cells have shed light on these issues. In mouse ESCs, it was confirmed that BMP4 significantly inhibits neural differentiation, as it does *in vivo* [28]. Moreover, the addition of a BMP antagonist was found to result in an obvious increase in the number of neural cells [29]. Therefore, the ESC neural differentiation system is an amenable model in which to study the functions and mechanisms of BMP signaling. BMPs have been implicated in maintaining the pluripotency of mouse ESCs through inducing the expression of inhibitor of differentiation (*Id*) genes to specifically block neural differentiation [30]. In addition to their roles in pluripotency maintenance, BMPs also induce mesodermal and epidermal differentiation in mouse ESCs [31–35]. The dual role of the BMP signal, to maintain ESC pluripotency and to induce non-neural differentiation, seems inconsistent. Recently, our studies in mouse ESCs and EpiSCs revealed that BMP signaling plays distinct roles during different stages of ESC neural differentiation [36]. We first found that cells at a specific period during mouse ESC neural differentiation mimic the late epiblast state and can be maintained as ESC-derived epiblast stem cells (ESD-EpiSCs). Thus, the ESC neural induction process can be divided into two stages: from ESCs to ESD-EpiSCs and from ESD-EpiSCs to neural progenitor cells. Using this system, it was revealed that BMP4 maintains ESC pluripotency by preventing cells from differentiating into late epiblast-like cells rather than by directly blocking ESC neural commitment. When the cells have been committed to becoming late epiblast cells, BMP4 cannot maintain their pluripotency, but it acts to inhibit neural induction by promoting mesodermal, epidermal and trophectodermal differentiation. Therefore, the late epiblast stage is the critical time point during which BMP4 switches its function from maintaining ESC pluripotency to promoting ESD-EpiSC non-neural differentiation.

Based on this model, the molecular basis of the distinct functions of BMP4 was further investigated [36]. *Ids*, the direct downstream targets of BMP, were found to inhibit the conversion of ESCs into ESD-EpiSCs during the first stage, and to reduce ESD-EpiSC self-renewal, inhibit neural specification and promote mesodermal and trophectodermal differentiation during the second stage. FGF-Erk signaling was also found to be involved in the functions of BMP signaling during different stages. In ESCs, FGF-Erk activity was found to be reduced by short-term treatment with BMP4, whereas in ESD-EpiSCs, FGF-Erk activity increased during long-term treatment with BMP4. Therefore, BMP might perform its stage-specific roles by interfering with the FGF-Erk pathway.

Due to the complexity of embryogenesis, the BMP signal may have divergent roles at other time points during the multi-step developmental process. For instance, BMP specifically induces an epidermal fate in ectoderm, which has been demonstrated in *Xenopus*. However, the ectoderm stage is usually overlooked during early embryonic development in chick and mouse because of its difficult accessibility and lack of markers. To confirm the default model of neural induction from ectoderm and the function of BMP during this process, an *in vitro* model of ectodermal cells needs to be established. We have recently identified ectoderm-like cells that form during mouse EpiSC neural and epidermal differentiation [37], and the signaling pathways involved in ectodermal cell commitment and neural differentiation are currently being investigated.

## 3. FGF Signaling

The studies reviewed above indicate a central role for BMP signaling in both the maintenance of mouse ESC pluripotency and neural inhibition. Data from different species show that the signaling of fibroblast growth factors (FGFs) also plays an important role during early embryogenesis through both BMP-dependent and -independent mechanisms. In *Xenopus*, it has been demonstrated that in addition to inhibition of BMP, pre-gastrula FGF signaling is also required in the ectoderm for the emergence of neural fates [38]. In chick embryos, the function of FGF in neural induction has been studied in detail. It was shown that inhibition of FGF signaling blocks neural induction [21]. Moreover, FGF initiates the onset of neural differentiation and suppresses BMP expression in the epiblast before gastrulation [19,21,39]. At later stages of embryogenesis, the major functions of FGF signaling are to induce mesoderm and to regulate movement during gastrulation [40,41]. FGFs control the specification and maintenance of mesoderm by regulating T box transcription factors in *Xenopus* [42,43], zebrafish [44,45] and mouse [46,47]. Thus, FGF signaling initiates the onset of neural differentiation in the epiblast before gastrulation, and it induces mesoderm formation and regulates gastrulation movements during later stages.

However, an important role for FGF signaling in neural induction has not been conclusively demonstrated in mice. Mutation of *Fgf4* or *Fgfr2* results in peri-implantation lethality [48,49], which suggests that FGF signaling is required very early during embryogenesis. Because of this early lethality, it is difficult to determine the role of FGF signaling in neural induction. *Fgf8* and *Fgfr1* are also expressed in the blastocyst, but they appear to function later. Both *Fgf8* mutants and *Fgfr1* mutants die at late gastrulation with impaired axis formation and mesoderm specification [50,51]. In *Fgf8*^−/−^ embryos, patterning of the prospective neuroectoderm is greatly perturbed and the range of anterior neuroectoderm markers is widely expanded [46]. Another study showed that inhibition of FGF signaling in E5.5-E6.5 mouse embryos leads to a drastic increase in the proportion of embryos displaying ectopic expression of the neural marker *Hesx1* [27]. Therefore, a positive effect of FGF in neural induction is not supported by the data from mouse embryos. Rather, FGF signaling negatively regulates the specification of neuroectoderm cell fate in post-implantation mouse embryos.

Thus, the function of FGF signaling in the pre-implantation mouse embryo needs to be elaborated. The answer to this question may explain the neural induction effect of FGF observed in *Xenopus* and chick. Recently, the early roles of FGF signaling have become increasingly understood using mouse pluripotent stem cells. In 2007, Stavridis *et al.* found that inhibition of FGF/Erk during mouse ESC differentiation abolished neuronal induction with no reduction in levels of the pluripotency marker *Nanog* [52]. Kunath *et al.* further showed that mouse ESCs lacking *Fgf4* or *Erk2* and those treated with FGFR inhibitors not only resist neural induction, but also fail to undergo mesodermal differentiation even when BMP4 is added. Moreover, *Erk2-*null ESCs retain their expression of the pluripotency markers *Oct4*, *Nanog* and *Rex1* when differentiated in adherent culture [53]. Therefore, when FGF signaling is inhibited, ESCs are deficient in the ability to commit to multiple lineages and stay in an undifferentiated state. These findings indicate that the FGF-Erk pathway primes ESCs for differentiation into a transitional stage that is analogous to the late epiblast state. It is possible that FGF signaling does not directly induce neural specification at the early stage of development but instead promotes ESCs to exit from self-renewal and enter a state in which they are more prepared for differentiation.

Subsequently, Ying *et al.* found that FGF-Erk inhibitors, in combination with the inhibition of glycogen synthase kinase 3 (GSK3; which promotes cellular growth and viability), keep mouse ESCs in an undifferentiated state called the “ground state” [54]. Based on this finding, it was predicted that it should be possible to capture true ESCs from epiblasts of other species by blocking the FGF-Erk and GSK3 pathways [55]. Following this line of reasoning, germline-competent rat ESCs have been successfully generated by combining LIF with FGF-Erk and GSK3 inhibition [56,57]. Recently, human ESCs with biological and epigenetic characteristics similar to those of mouse ESCs were created by ectopic induction of Oct4, Klf4 and Klf2 combined with LIF and inhibition of the FGF-Erk and GSK3 pathways [58]. These data suggested that inhibition of FGF signaling blocks the transition of ESCs to a differentiation state. However, there was no direct evidence to suggest that this transition state is the late epiblast state. With the derivation of EpiSCs from mouse ESCs, this question could be resolved. In our recent report, we showed that either FGF4 or FGF2 can significantly increase the number of ESD-EpiSC colonies derived from differentiated ESC aggregates and that inhibition of FGF-Erk activity dramatically reduces the number of ESD-EpiSCs. Moreover, FGF2 partially counteracts the BMP4-induced reduction in ESD-EpiSC colony numbers [36]. These results strongly suggest an important role for FGF in the derivation of EpiSCs from ESCs. Recently, another group also reported the creation of an ESC-based system for isolating epiblast cells that are similar to EpiSCs, through which they confirmed that FGF signaling is crucial for priming ESCs to differentiate into the late epiblast state [59].

In addition to the early roles of FGF, the subsequent functions of FGF signaling starting from the late epiblast stage are being investigated in EpiSCs. First, as in human ESCs, FGF2 is required to maintain EpiSC pluripotency [12,13]. However, the mechanism through which FGF2 stabilizes the pluripotent state is different between these two cell types [60]. In human ESCs, FGF signaling in cooperation with SMAD2/3 signaling mediates NANOG expression, thereby actively promoting ESC self-renewal. In EpiSCs, however, FGF2 fails to regulate NANOG expression. Rather, it supports the epiblast state by inhibiting neuroectodermal induction, particularly by blocking *Pax6* expression [60]. Blockage of FGF signaling in EpiSCs promotes rapid neural induction and subsequent neurogenesis [59]. These results are consistent with previous findings in mutant mice that FGF negatively regulates the specification of neuroectodermal cell fate in post-implantation mouse embryos [27,46,50,51,61,62].

In summary, FGF signaling plays distinct roles during different developmental stages. FGF signaling is crucial for priming ESCs to differentiate into the late epiblast state. It then acts to inhibit the subsequent transition to neuroectoderm. This conclusion is distinct from the notion that neural differentiation is promoted by FGF signaling. In the future, it will be interesting to determine the exact functions of divergent FGF ligands during commitment to the three germ layers from the epiblast and to elucidate the molecular mechanisms underlying these processes using EpiSCs.

## 4. Nodal Signaling

Nodal, a member of the TGF-β family, plays central roles in early embryo patterning during the induction of mesoderm and endoderm and the specification of left-right asymmetry. In some cases, Nodal signaling is also referred to as “Activin/Nodal” signaling because another TGF-β family member, Activin, binds to the same receptors as Nodal (with the exception of the coreceptor Cripto) and triggers similar intracellular events [63].

The roles of Nodal signaling are evolutionarily conserved and have been well established using molecular genetic studies in various animal models. In *Xenopus*, Activin and Xnrs (*Xenopus* homologues of Nodal) function as mesoderm inducers in whole embryos and explanted animal caps [64–66]. Inhibition of Nodal signaling was found to block the formation of mesoderm and endoderm [67–69]. Genetic studies in zebrafish and mouse have also provided strong evidence that Nodal signaling is essential for mesendoderm formation. Zebrafish that are double mutants for *Cyclops* and *Squint*, the orthologs of *Nodal*, lack head and trunk mesoderm and fail to form the germ-ring, an organizer analogous to the mouse primitive streak [70]. Similarly, mice lacking Nodal lose the primitive streak and most mesoderm [71]. In addition, Nodal signaling is essential for the formation and patterning of the anterior visceral endoderm (AVE) in post-implantation mouse embryos [72]. AVE produces the Nodal antagonists Lefty1 and Cerberus-1 (Cer1) to limit the extension of primitive streaks and to maintain the correct patterning of the epiblast [73]. Although additional signals such as BMP and FGF are also involved in gastrulation, genetic evidence suggests that Nodal signals are core players in the formation of the primitive streak and in epiblast patterning.

Nodal signals may also act as anti-differentiation signals to maintain epiblast proliferation. Analysis of Nodal mutants and embryo explants suggests that Nodal signaling within the epiblast is essential for maintaining pluripotency determinants such as Oct4 and Nanog and for preventing precocious neuroectoderm differentiation [74,75]. *In vitro* studies have also confirmed that Nodal signaling is required for the maintenance of undifferentiated human ESCs and mouse EpiSCs [12,76]. Activin/Nodal signaling through SMAD2/3 activation is required to sustain the self-renewal of these pluripotent stem cells. It has been shown that SMAD2/3 binds to the *NANOG* promoter and thereby activates *NANOG* gene transcription in human ESCs [77,78] and mouse EpiSCs [60]. These results are consistent with findings in mouse embryos that Nodal is required to maintain epiblast pluripotency. Moreover, *in vitro* studies have revealed the corresponding molecular mechanism underlying this function.

In mouse ESCs, EpiSCs and human ESCs, Activin/Nodal signaling is also known to drive differentiation toward mesendoderm [14,79–81]. The functions of Activin/Nodal signaling in pluripotency maintenance and mesendoderm induction seem contradictory. Recently, Chng *et al.* provided an explanation for how Activin/Nodal signaling maintains pluripotency without inducing mesendoderm in human ESCs and mouse EpiSCs [82]. They found that Smad-interacting protein 1 (SIP1) has an essential role in the promotion of neuroectoderm differentiation and the suppression of mesendodermal genes induced by Activin/Nodal signaling. In turn, Activin/Nodal signaling cooperates with NANOG, OCT4 and SOX2 to control the expression of *SIP1* in human ESCs, thereby limiting the neuroectoderm promoting effects of SIP1. Therefore, SIP1 limits the mesendoderm inducing effects of Activin/Nodal signaling without inhibiting the pluripotency maintaining effects exerted by SMAD2/3. This conclusion was confirmed in mouse EpiSCs, implying that these mechanisms are conserved in different species and may operate *in vivo* during mammalian development [82]. Overall, there are still many unresolved questions about how Activin/Nodal signaling switches its function from pluripotency maintenance to mesendoderm induction. Future studies should focus on revealing the full molecular cascades by which Nodal signaling controls these cell-fate choices.

## 5. Wnt Signaling

Wnt signaling is mediated by blocking the activity of glycogen synthase kinase 3β (GSK3β), which promotes β-catenin degradation. Inhibition of Gsk3β activity thus stabilizes β-catenin, which activates the Wnt pathway. Wnt signaling plays multiple roles during early embryonic development. In both *Xenopus* and chick embryos, Wnt signaling represses neural fate and induces epidermal fate by attenuating the responsiveness of epiblast cells to FGF signaling [83–86]. In addition, Wnt signaling is required for primitive streak formation in chick and mouse embryos [87,88]. Conversely, a study in *Xenopus* found that Wnt signaling promotes neural development in ectoderm through inhibition of BMP4 expression [89]. Furthermore, *Xiro1*, a downstream target of the Wnt pathway, was shown to repress BMP4 expression [90]. These contradictory findings might be due to the different developmental stages during which the experiments were conducted. At early stages of development, Wnt signaling is activated on the dorsal side of the embryo, where it represses the activity of BMP signaling and specifies the dorsal character. Consequently, cells on the dorsal side give rise to neural cells [89]. However, at the blastula/early gastrula stages, Wnt signaling may suppress the generation of neural cells [86]. Therefore, it becomes necessary to inhibit Wnt signaling for normal neural cell development at later stages [91].

The functions and mechanisms of Wnt signaling have also been investigated in mouse ESCs. In agreement with observations *in vivo*, Wnt signaling is required for mesoderm differentiation in mouse ESCs. It has been shown that inhibition of endogenous Wnt signals in mouse ESCs prevents the expression of primitive streak-, endoderm- and mesoderm-associated genes and abrogates the functional development of mature mesodermal lineages [92]. However, β-catenin alone is not sufficient to promote primitive streak-associated gene expression, indicating that Wnt/β-catenin may cooperate with other signals to regulate germ layer induction. Another study in mouse ESCs showed that Wnt and Nodal signaling are required to act together for the formation of mesendodermal cells [10]. In summary, Wnt signaling cooperates with other signaling pathways to regulate mesoderm and endoderm differentiation in mouse ESCs.

Recently, Sato *et al.* showed that Wnt activation stimulated by 6-bromoindirubin-3′-oxime (BIO), a specific pharmacological inhibitor of GSK3, sustains the expression of the pluripotent state-specific transcription factors Oct4, Rex1 and Nanog in mouse and human ESCs [93]. In addition, Doble *et al.* reported that overexpression of stabilized β-catenin in mouse ESCs inhibits neuronal differentiation and delays loss of pluripotency; moreover, β-catenin forms a complex with Oct4 and enhances Oct4 activity [94]. Therefore, Wnt/β-catenin signaling plays a role, in part through its interaction with Oct4, in the maintenance of pluripotency. However, Wnt signaling alone is not sufficient to maintain the ground state of mouse ESCs. Ying *et al.* showed that blockage of GSK3 in mouse ESCs enhances growth capacity and suppresses neural differentiation, but it also promotes non-neural differentiation. To block differentiation of ESCs into the cells that make up the three germ lineages, the combination of a GSK3 inhibitor and an FGF-Erk inhibitor is necessary [54].

At present, no published data fully elucidate the functions of Wnt signaling in EpiSC differentiation. Considering the multiple roles of Wnt signaling in early embryonic development, we speculate that Wnt signaling may play distinct roles at different stages of development, similarly to the BMP, FGF and Nodal pathways. Further investigation into the functions of Wnt and its crosstalk with other signaling pathways is needed, especially in EpiSCs.

## 6. Outlook

The BMP, FGF, Nodal and Wnt signaling pathways play important roles during embryogenesis. However, the mechanisms underlying cell fate decisions during vertebrate embryogenesis are complex. ESCs and EpiSCs provide *in vitro* systems for investigating the complex mechanisms through which signaling pathways play distinct roles during different developmental stages. Recently, published data [95] and our unpublished results suggest that it may be possible to derive ectodermal cells directly from mouse ESCs and EpiSCs. Ectodermal cells would work as a unique *in vitro* tool to study the mechanisms involved in ectoderm commitment and in neural and epithelial differentiation during embryonic development. Studies in pluripotent stem cells that correspond to different developmental stages would provide a foundation for efforts to guide the differentiation of pluripotent stem cells along selected developmental pathways for potential therapeutic use.

## References

[1] Gardner R.L., Rossant J. (1979). Investigation of the fate of 4–5 day post-coitum mouse inner cell mass cells by blastocyst injection. J. Embryol. Exp. Morphol..

[2] Coucouvanis E., Martin G.R. (1995). Signals for death and survival: A two-step mechanism for cavitation in the vertebrate embryo. Cell.

[3] Lu C.C., Brennan J., Robertson E.J. (2001). From fertilization to gastrulation: axis formation in the mouse embryo. Curr. Opin. Genet. Dev..

[4] Evans M.J., Kaufman M.H. (1981). Establishment in culture of pluripotential cells from mouse embryos. Nature.

[5] Martin G.R. (1981). Isolation of a pluripotent cell line from early mouse embryos cultured in medium conditioned by teratocarcinoma stem cells. Proc. Natl. Acad. Sci. USA.

[6] Thomson J.A., Itskovitz-Eldor J., Shapiro S.S., Waknitz M.A., Swiergiel J.J., Marshall V.S., Jones J.M. (1998). Embryonic stem cell lines derived from human blastocysts. Science.

[7] Reubinoff B.E., Pera M.F., Fong C.Y., Trounson A., Bongso A. (2000). Embryonic stem cell lines from human blastocysts: Somatic differentiation in vitro. Nat. Biotechnol..

[8] Schuldiner M., Yanuka O., Itskovitz-Eldor J., Melton D.A., Benvenisty N. (2000). Effects of eight growth factors on the differentiation of cells derived from human embryonic stem cells. Proc. Natl. Acad. Sci. USA.

[9] Odorico J.S., Kaufman D.S., Thomson J.A. (2001). Multilineage differentiation from human embryonic stem cell lines. Stem Cells.

[10] Gadue P., Huber T.L., Paddison P.J., Keller G.M. (2006). Wnt and TGF-beta signaling are required for the induction of an in vitro model of primitive streak formation using embryonic stem cells. Proc. Natl. Acad. Sci. USA.

[11] Sulzbacher S., Schroeder I.S., Truong T.T., Wobus A.M. (2009). Activin A-induced differentiation of embryonic stem cells into endoderm and pancreatic progenitors-the influence of differentiation factors and culture conditions. Stem Cell Rev..

[12] Brons I.G., Smithers L.E., Trotter M.W., Rugg-Gunn P., Sun B., Chuva de Sousa Lopes S.M., Howlett S.K., Clarkson A., Ahrlund-Richter L., Pedersen R.A., Vallier L. (2007). Derivation of pluripotent epiblast stem cells from mammalian embryos. Nature.

[13] Tesar P.J., Chenoweth J.G., Brook F.A., Davies T.J., Evans E.P., Mack D.L., Gardner R.L., McKay R.D. (2007). New cell lines from mouse epiblast share defining features with human embryonic stem cells. Nature.

[14] Vallier L., Touboul T., Chng Z., Brimpari M., Hannan N., Millan E., Smithers L.E., Trotter M., Rugg-Gunn P., Weber A., Pedersen R.A. (2009). Early cell fate decisions of human embryonic stem cells and mouse epiblast stem cells are controlled by the same signalling pathways. PLoS One.

[15] Wilson P.A., Hemmati-Brivanlou A. (1995). Induction of epidermis and inhibition of neural fate by Bmp-4. Nature.

[16] Wilson P.A., Hemmati-Brivanlou A. (1997). Vertebrate neural induction: inducers, inhibitors, and a new synthesis. Neuron.

[17] Wilson S.I., Edlund T. (2001). Neural induction: toward a unifying mechanism. Nat. Neurosci..

[18] Streit A., Lee K.J., Woo I., Roberts C., Jessell T.M., Stern C.D. (1998). Chordin regulates primitive streak development and the stability of induced neural cells, but is not sufficient for neural induction in the chick embryo. Development.

[19] Wilson S.I., Graziano E., Harland R., Jessell T.M., Edlund T. (2000). An early requirement for FGF signalling in the acquisition of neural cell fate in the chick embryo. Curr. Biol..

[20] Linker C., Stern C.D. (2004). Neural induction requires BMP inhibition only as a late step, and involves signals other than FGF and Wnt antagonists. Development.

[21] Streit A., Berliner A.J., Papanayotou C., Sirulnik A., Stern C.D. (2000). Initiation of neural induction by FGF signalling before gastrulation. Nature.

[22] Stern C.D. (2006). Neural induction: 10 years on since the ‘default model’. Curr. Opin. Cell Biol..

[23] Bachiller D., Klingensmith J., Kemp C., Belo J.A., Anderson R.M., May S.R., McMahon J.A., McMahon A.P., Harland R.M., Rossant J., De Robertis E.M. (2000). The organizer factors Chordin and Noggin are required for mouse forebrain development. Nature.

[24] Anderson R.M., Lawrence A.R., Stottmann R.W., Bachiller D., Klingensmith J. (2002). Chordin and noggin promote organizing centers of forebrain development in the mouse. Development.

[25] Bachiller D., Klingensmith J., Shneyder N., Tran U., Anderson R., Rossant J., De Robertis E.M. (2003). The role of chordin/Bmp signals in mammalian pharyngeal development and DiGeorge syndrome. Development.

[26] Mishina Y., Suzuki A., Ueno N., Behringer R.R. (1995). Bmpr encodes a type I bone morphogenetic protein receptor that is essential for gastrulation during mouse embryogenesis. Genes. Dev..

[27] Di-Gregorio A., Sancho M., Stuckey D.W., Crompton L.A., Godwin J., Mishina Y., Rodriguez T.A. (2007). BMP signalling inhibits premature neural differentiation in the mouse embryo. Development.

[28] Finley M.F., Devata S., Huettner J.E. (1999). BMP-4 inhibits neural differentiation of murine embryonic stem cells. J. Neurobiol..

[29] Tropepe V., Hitoshi S., Sirard C., Mak T.W., Rossant J., van der Kooy D. (2001). Direct neural fate specification from embryonic stem cells: A primitive mammalian neural stem cell stage acquired through a default mechanism. Neuron.

[30] Ying Q.L., Nichols J., Chambers I., Smith A. (2003). BMP induction of Id proteins suppresses differentiation and sustains embryonic stem cell self-renewal in collaboration with STAT3. Cell.

[31] Park C., Afrikanova I., Chung Y.S., Zhang W.J., Arentson E., Fong Gh G., Rosendahl A., Choi K. (2004). A hierarchical order of factors in the generation of FLK1- and SCL-expressing hematopoietic and endothelial progenitors from embryonic stem cells. Development.

[32] Pearson S., Sroczynska P., Lacaud G., Kouskoff V. (2008). The stepwise specification of embryonic stem cells to hematopoietic fate is driven by sequential exposure to Bmp4, activin A, bFGF and VEGF. Development.

[33] Metallo C.M., Ji L., de Pablo J.J., Palecek S.P. (2008). Retinoic acid and bone morphogenetic protein signaling synergize to efficiently direct epithelial differentiation of human embryonic stem cells. Stem Cells.

[34] Aberdam D., Gambaro K., Rostagno P., Aberdam E., de la Forest Divonne S., Rouleau M. (2007). Key role of p63 in BMP-4-induced epidermal commitment of embryonic stem cells. Cell Cycle.

[35] Haase I., Knaup R., Wartenberg M., Sauer H., Hescheler J., Mahrle G. (2007). In vitro differentiation of murine embryonic stem cells into keratinocyte-like cells. Eur. J Cell Biol..

[36] Zhang K., Li L., Huang C., Shen C., Tan F., Xia C., Liu P., Rossant J., Jing N. (2010). Distinct functions of BMP4 during different stages of mouse ES cell neural commitment. Development.

[b37-genes-02-00298] 37.Li, L.Y.; Jing, N.H. Unpublished work, 2010.

[38] Delaune E., Lemaire P., Kodjabachian L. (2005). Neural induction in Xenopus requires early FGF signalling in addition to BMP inhibition. Development.

[39] Sheng G., dos Reis M., Stern C.D. (2003). Churchill, a zinc finger transcriptional activator, regulates the transition between gastrulation and neurulation. Cell.

[40] Sivak J.M., Petersen L.F., Amaya E. (2005). FGF signal interpretation is directed by Sprouty and Spred proteins during mesoderm formation. Dev. Cell.

[41] Fletcher R.B., Baker J.C., Harland R.M. (2006). FGF8 spliceforms mediate early mesoderm and posterior neural tissue formation in Xenopus. Development.

[42] Smith J.C., Price B.M., Green J.B., Weigel D., Herrmann B.G. (1991). Expression of a Xenopus homolog of Brachyury (T) is an immediate-early response to mesoderm induction. Cell.

[43] Strong C.F., Barnett M.W., Hartman D., Jones E.A., Stott D. (2000). Xbra3 induces mesoderm and neural tissue in Xenopus laevis. Dev. Biol..

[44] Griffin K.J., Amacher S.L., Kimmel C.B., Kimelman D. (1998). Molecular identification of spadetail: regulation of zebrafish trunk and tail mesoderm formation by T-box genes. Development.

[45] Zhao J., Cao Y., Zhao C., Postlethwait J., Meng A. (2003). An SP1-like transcription factor Spr2 acts downstream of Fgf signaling to mediate mesoderm induction. EMBO J..

[46] Sun X., Meyers E.N., Lewandoski M., Martin G.R. (1999). Targeted disruption of Fgf8 causes failure of cell migration in the gastrulating mouse embryo. Genes Dev..

[47] Ciruna B., Rossant J. (2001). FGF signaling regulates mesoderm cell fate specification and morphogenetic movement at the primitive streak. Dev. Cell.

[48] Arman E., Haffner-Krausz R., Chen Y., Heath J.K., Lonai P. (1998). Targeted disruption of fibroblast growth factor (FGF) receptor. 2 suggests a role for FGF signaling in pregastrulation mammalian development. Proc. Natl. Acad. Sci. USA.

[49] Feldman B., Poueymirou W., Papaioannou V.E., DeChiara T.M., Goldfarb M. (1995). Requirement of FGF-4 for postimplantation mouse development. Science.

[50] Deng C.X., Wynshaw-Boris A., Shen M.M., Daugherty C., Ornitz D.M., Leder P. (1994). Murine FGFR-1 is required for early postimplantation growth and axial organization. Genes. Dev..

[51] Meyers E.N., Lewandoski M., Martin G.R. (1998). An Fgf8 mutant allelic series generated by Cre- and Flp-mediated recombination. Nat. Genet..

[52] Stavridis M.P., Lunn J.S., Collins B.J., Storey K.G. (2007). A discrete period of FGF-induced Erk1/2 signalling is required for vertebrate neural specification. Development.

[53] Kunath T., Saba-El-Leil M.K., Almousailleakh M., Wray J., Meloche S., Smith A. (2007). FGF stimulation of the Erk1/2 signalling cascade triggers transition of pluripotent embryonic stem cells from self-renewal to lineage commitment. Development.

[54] Ying Q.L., Wray J., Nichols J., Batlle-Morera L., Doble B., Woodgett J., Cohen P., Smith A. (2008). The ground state of embryonic stem cell self-renewal. Nature.

[55] Silva J., Smith A. (2008). Capturing pluripotency. Cell.

[56] Buehr M., Meek S., Blair K., Yang J., Ure J., Silva J., McLay R., Hall J., Ying Q.L., Smith A. (2008). Capture of authentic embryonic stem cells from rat blastocysts. Cell.

[57] Li P., Tong C., Mehrian-Shai R., Jia L., Wu N., Yan Y., Maxson R.E., Schulze E.N., Song H., Hsieh C.L., Pera M.F., Ying Q.L. (2008). Germline competent embryonic stem cells derived from rat blastocysts. Cell.

[58] Hanna J., Cheng A.W., Saha K., Kim J., Lengner C.J., Soldner F., Cassady J.P., Muffat J., Carey B.W., Jaenisch R. (2010). Human embryonic stem cells with biological and epigenetic characteristics similar to those of mouse ESCs. Proc. Natl. Acad. Sci. USA.

[59] Sterneckert J., Stehling M., Bernemann C., Arauzo-Bravo M.J., Greber B., Gentile L., Ortmeier C., Sinn M., Wu G., Ruau D., Zenke M., Brintrup R., Klein D.C., Ko K., Scholer H.R. (2010). Neural induction intermediates exhibit distinct roles of Fgf signaling. Stem Cells..

[60] Greber B., Wu G., Bernemann C., Joo J.Y., Han D.W., Ko K., Tapia N., Sabour D., Sterneckert J., Tesar P., Scholer H.R. (2010). Conserved and divergent roles of FGF signaling in mouse epiblast stem cells and human embryonic stem cells. Cell Stem Cell.

[61] Ciruna B.G., Schwartz L., Harpal K., Yamaguchi T.P., Rossant J. (1997). Chimeric analysis of fibroblast growth factor receptor-1 (Fgfr1) function: a role for FGFR1 in morphogenetic movement through the primitive streak. Development.

[62] Deng C., Bedford M., Li C., Xu X., Yang X., Dunmore J., Leder P. (1997). Fibroblast growth factor receptor-1 (FGFR-1) is essential for normal neural tube and limb development. Dev. Biol..

[63] de Caestecker M. (2004). The transforming growth factor-beta superfamily of receptors. Cytokine Growth Factor Rev..

[64] Harland R., Gerhart J. (1997). Formation and function of Spemann's organizer. Annu. Rev. Cell Dev. Biol..

[65] Jones C.M., Kuehn M.R., Hogan B.L., Smith J.C., Wright C.V. (1995). Nodal-related signals induce axial mesoderm and dorsalize mesoderm during gastrulation. Development.

[66] Joseph E.M., Melton D.A. (1997). Xnr4: A Xenopus nodal-related gene expressed in the Spemann organizer. Dev. Biol..

[67] Osada S.I., Wright C.V. (1999). Xenopus nodal-related signaling is essential for mesendodermal patterning during early embryogenesis. Development.

[68] Agius E., Oelgeschlager M., Wessely O., Kemp C., De Robertis E.M. (2000). Endodermal Nodal-related signals and mesoderm induction in Xenopus. Development.

[69] Cheng A.M., Thisse B., Thisse C., Wright C.V. (2000). The lefty-related factor Xatv acts as a feedback inhibitor of nodal signaling in mesoderm induction and L-R axis development in xenopus. Development.

[70] Feldman B., Gates M.A., Egan E.S., Dougan S.T., Rennebeck G., Sirotkin H.I., Schier A.F., Talbot W.S. (1998). Zebrafish organizer development and germ-layer formation require nodal-related signals. Nature.

[71] Conlon F.L., Lyons K.M., Takaesu N., Barth K.S., Kispert A., Herrmann B., Robertson E.J. (1994). A primary requirement for nodal in the formation and maintenance of the primitive streak in the mouse. Development.

[72] Brennan J., Lu C.C., Norris D.P., Rodriguez T.A., Beddington R.S., Robertson E.J. (2001). Nodal signalling in the epiblast patterns the early mouse embryo. Nature.

[73] Perea-Gomez A., Vella F.D., Shawlot W., Oulad-Abdelghani M., Chazaud C., Meno C., Pfister V., Chen L., Robertson E., Hamada H., Behringer R.R., Ang S.L. (2002). Nodal antagonists in the anterior visceral endoderm prevent the formation of multiple primitive streaks. Dev. Cell.

[74] Mesnard D., Guzman-Ayala M., Constam D.B. (2006). Nodal specifies embryonic visceral endoderm and sustains pluripotent cells in the epiblast before overt axial patterning. Development.

[75] Camus A., Perea-Gomez A., Moreau A., Collignon J. (2006). Absence of Nodal signaling promotes precocious neural differentiation in the mouse embryo. Dev. Biol..

[76] Brandenberger R., Wei H., Zhang S., Lei S., Murage J., Fisk G.J., Li Y., Xu C., Fang R., Guegler K., Rao M.S., Mandalam R., Lebkowski J., Stanton L.W. (2004). Transcriptome characterization elucidates signaling networks that control human ES cell growth and differentiation. Nat. Biotechnol..

[77] Xu R.H., Sampsell-Barron T.L., Gu F., Root S., Peck R.M., Pan G., Yu J., Antosiewicz-Bourget J., Tian S., Stewart R., Thomson J.A. (2008). NANOG is a direct target of TGFbeta/activin-mediated SMAD signaling in human ESCs. Cell Stem Cell.

[78] Greber B., Lehrach H., Adjaye J. (2008). Control of early fate decisions in human ES cells by distinct states of TGFbeta pathway activity. Stem. Cells. Dev..

[79] Tada S., Era T., Furusawa C., Sakurai H., Nishikawa S., Kinoshita M., Nakao K., Chiba T. (2005). Characterization of mesendoderm: A diverging point of the definitive endoderm and mesoderm in embryonic stem cell differentiation culture. Development.

[80] Takenaga M., Fukumoto M., Hori Y. (2007). Regulated Nodal signaling promotes differentiation of the definitive endoderm and mesoderm from ES cells. J. Cell Sci..

[81] D'Amour K.A., Agulnick A.D., Eliazer S., Kelly O.G., Kroon E., Baetge E.E. (2005). Efficient differentiation of human embryonic stem cells to definitive endoderm. Nat. Biotechnol..

[82] Chng Z., Teo A., Pedersen R.A., Vallier L. (2010). SIP1 mediates cell-fate decisions between neuroectoderm and mesendoderm in human pluripotent stem cells. Cell Stem Cell.

[83] Glinka A., Wu W., Onichtchouk D., Blumenstock C., Niehrs C. (1997). Head induction by simultaneous repression of Bmp and Wnt signalling in Xenopus. Nature.

[84] Glinka A., Wu W., Delius H., Monaghan A.P., Blumenstock C., Niehrs C. (1998). Dickkopf-1 is a member of a new family of secreted proteins and functions in head induction. Nature.

[85] Itoh K., Tang T.L., Neel B.G., Sokol S.Y. (1995). Specific modulation of ectodermal cell fates in Xenopus embryos by glycogen synthase kinase. Development.

[86] Wilson S.I., Rydstrom A., Trimborn T., Willert K., Nusse R., Jessell T.M., Edlund T. (2001). The status of Wnt signalling regulates neural and epidermal fates in the chick embryo. Nature.

[87] Skromne I., Stern C.D. (2001). Interactions between Wnt and Vg1 signalling pathways initiate primitive streak formation in the chick embryo. Development.

[88] Liu P., Wakamiya M., Shea M.J., Albrecht U., Behringer R.R., Bradley A. (1999). Requirement for Wnt3 in vertebrate axis formation. Nat. Genet..

[89] Baker J.C., Beddington R.S., Harland R.M. (1999). Wnt signaling in Xenopus embryos inhibits bmp4 expression and activates neural development. Genes. Dev..

[90] Gomez-Skarmeta J., de La Calle-Mustienes E., Modolell J. (2001). The Wnt-activated Xiro1 gene encodes a repressor that is essential for neural development and downregulates Bmp4. Development.

[91] Stern C.D. (2005). Neural induction: old problem, new findings, yet more questions. Development.

[92] Lindsley R.C., Gill J.G., Kyba M., Murphy T.L., Murphy K.M. (2006). Canonical Wnt signaling is required for development of embryonic stem cell-derived mesoderm. Development.

[93] Sato N., Meijer L., Skaltsounis L., Greengard P., Brivanlou A.H. (2004). Maintenance of pluripotency in human and mouse embryonic stem cells through activation of Wnt signaling by a pharmacological GSK-3-specific inhibitor. Nat. Med..

[94] Kelly K.F., Ng D.Y., Jayakumaran G., Wood G.A., Koide H., Doble B.W. (2011). beta-Catenin Enhances Oct-4 Activity and Reinforces Pluripotency through a TCF-Independent Mechanism. Cell Stem Cell.

[95] Harvey N.T., Hughes J.N., Lonic A., Yap C., Long C., Rathjen P.D., Rathjen J. (2010). Response to BMP4 signalling during ES cell differentiation defines intermediates of the ectoderm lineage. J. Cell Sci..

